# The Efficacy and Medium to Long-Term Follow-Up of Transcatheter Retrograde Closure of Perimembranous Ventricular Septal Defects via the Femoral Artery With Amplatzer Duct Occluder II in Children

**DOI:** 10.3389/fped.2021.571407

**Published:** 2021-05-25

**Authors:** Diandong Jiang, Jianjun Zhang, Youfei Fan, Bo Han, Lijian Zhao, Yingchun Yi, Jianli Lv, Jing Wang

**Affiliations:** Department of Pediatric Cardiology, Shandong Provincial Hospital Affiliated to Shandong First Medical University, Jinan, China

**Keywords:** Amplatzer Duct Occluder II, ventricular septal defect, transcatheter closure, retrograde, follow-up

## Abstract

**Objective:** The aim of this study was to assess the efficacy and safety of transcatheter retrograde closure of perimembranous ventricular septal defect (pmVSD) via the femoral artery with an Amplatzer Duct Occluder II (ADO II) in children.

**Methods:** The clinical and follow-up data of 102 children who had successfully undergone transcatheter retrograde closure of pmVSD via the femoral artery with Amplatzer Duct Occluder II (ADO II) from February 2012 to June 2019 in our center were retrospectively reviewed.

**Results:** In 102 of 103 patients, the defects were successfully closed (99.0%). The average age was 4.03 ± 1.84 years and the mean weight was 17.50 ± 5.58 kg. The mean diameter of the defects was 2.58 ± 0.63 mm. Hundred of the occluders had a waist length of 4 mm. The complete closure rate was 89.2% 24 h after the procedure and 98% at the last follow-up. The follow-up period ranged from 6 to 92 months, with a median of 36 months. One child developed new mild aortic regurgitation (AR), and 9 patients developed new mild tricuspid regurgitation (TR). During follow-up, TR disappeared in 5 patients and decreased in 4 patients, and AR relieved in one patient. One patient developed intermittent complete left bundle branch block 4 days after the procedure and recovered sinus rhythm 2 days later. No serious complications occurred.

**Conclusion:** Transcatheter retrograde closure of pmVSD via the femoral artery with ADO II in children is safe, feasible, and effective in selected patients.

## Introduction

Ventricular septal defect (VSD) is the most common congenital heart disease (CHD), accounting for ~20% of all CHDs (1). Depending on their location within the septum, the defects can be divided into perimembranous, muscular and supracristal, with perimembranous VSD (pmVSD) constituting about 60–70%. Currently, percutaneous closure of pmVSD is widely performed especially in Asian countries. Post-procedure heart block especially complete atrioventricular block (cAVB) has always been one of the major concerns in the transcatheter closure of pmVSD. Previous literatures (2, 3) have described that heart block is closely related to the selection of occluders.

In recent years, closure of pmVSD using Amplatzer Duct Occluder II (ADO II) has been gradually reported. The overall efficacy is encouraging with acceptably low complications (4–6). Nevertheless, researches about this approach for pmVSD closure have been limited to small series with deficient follow-up up to now. In closure of pmVSD, most of ADO II were released through the anterograde insertion route via the femoral vein and fewer through the retrograde route via the femoral artery. Since 2012, our center started to perform transcatheter retrograde closure of pmVSD via the femoral artery using ADO II. To our knowledge, our study includes the largest series of pediatric patients whose pmVSD was closed using ADO II through the retrograde route via the femoral artery. The purpose of this study was to assess the efficacy and safety of transcatheter retrograde closure of pmVSD through retrospective analysis of the experience and medium- to long-term follow-up data.

## Patients and Methods

From February 2012 to June 2019, 870 pediatric patients with pmVSD were enrolled for attempted transcatheter closure in our center, and a total of 102 children who had successfully undergone transcatheter retrograde closure via the femoral artery with ADO II were retrospectively reviewed. All patients were diagnosed by transthoracic echocardiography (TTE) before the procedure. Informed written consent for the procedure was given by the guardians of the children before the procedure (general patient data are shown in Table 1).

**Table 1:** General patient data.

**Variables**	**Values**
**Sex**	
Male	52
Female	50
**Age**	
<3 years	23 (22.5%)
3–6 years	72 (70.6%)
>6 years	7 (6.9%)
Weight	17.50 ± 5.58 kg (11–50)
<15 kg	32 (31.4%)
15–20 kg	50 (49.0%)
>20 kg	20 (19.6%)
**Perimembranous aneurysm**	
Without	10 (9.8%)
With	92 (90.2%)

*Values are n (%) or mean ± SD*.

Inclusion Criteria:

1. Age ≥ 2 years old, weight ≥ 10 kg;
2. Clinical symptoms or left ventricular overload performance;

Clinical symptoms: Growth retardation, recurrent respiratory infections, and heart failure not improving with medications;

Left ventricular overload performance: Electrocardiogram (ECG) showing left atrial enlargement and left ventricular hypertrophy, cardiothoracic ratio on chest X-ray of > 0.5, TTE showing significant left to right shunt with left atrium and left ventricular enlargement (Left ventricular enlargement, defined as left ventricular end-diastolic z-score on echocardiogram, indexed to body surface area of >2.0);

3. The selection of ADO II based on left ventricular angiography:

Defect size: Defect diameter (left ventricular inlet or right ventricular outlet) <6 mm, the distance among multiple outlets <6 mm;

The distance of the defect to the aortic valve: The distance from the upper edge of inlet or outlet to the aortic valve > 3 mm.

Exclusion Criteria:

1. Severe pulmonary hypertension with bidirectional shunt or right to left shunt;
2. VSD associated with other structural heart defects requiring surgery;
3. Defect diameter > 6 mm, or the distance among multiple outlets > 6 mm;
4. The distance from the upper edge of inlet and outlet to the aortic valve <3 mm.

### Catheterization Procedure

**Figure 1:**
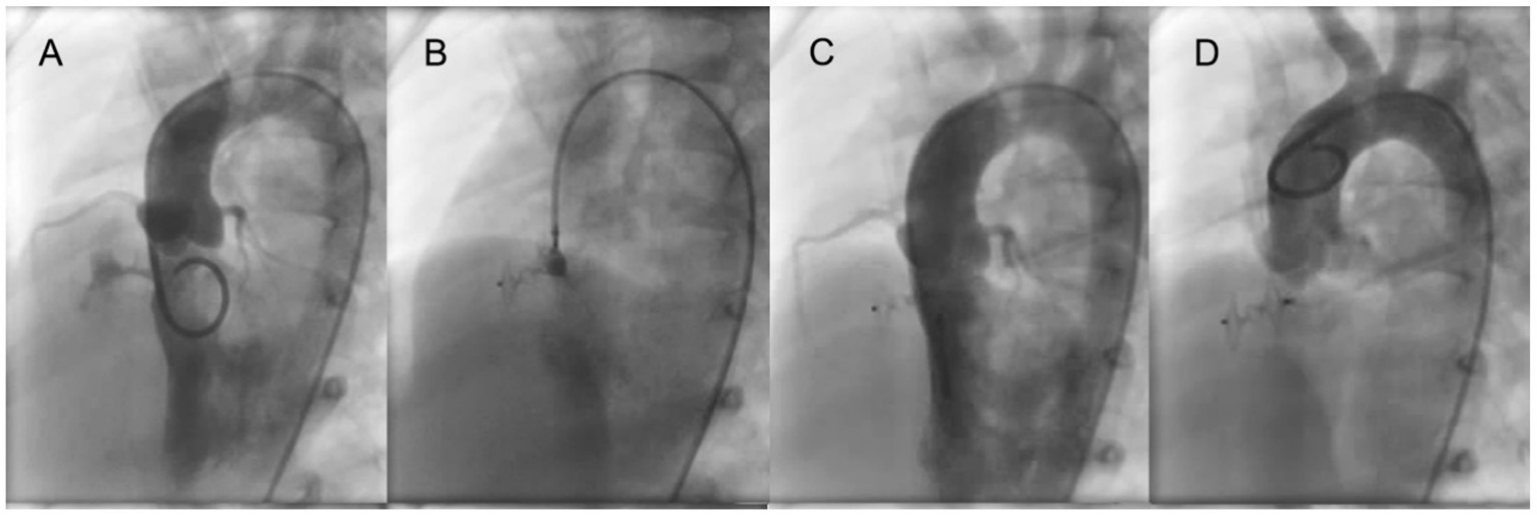
ADO II is used to close a tubular pmVSD. **(A)** Left ventricular angiography shows a long shunt jet with tubular shape. **(B)** Before occluder release, the left disk is pressed into the left ventricle through the delivery sheath and left ventricular angiography is performed to confirm that the left disc is under the aortic valve. Also, transthoracic echocardiography is performed to determine if the left disc is causing aortic regurgitation. **(C)** Left ventricular angiography after occluder release. **(D)** Ascending aorta angiography after occluder release. pmVSD, perimembranous ventricular septal defect.

**Figure 2:**
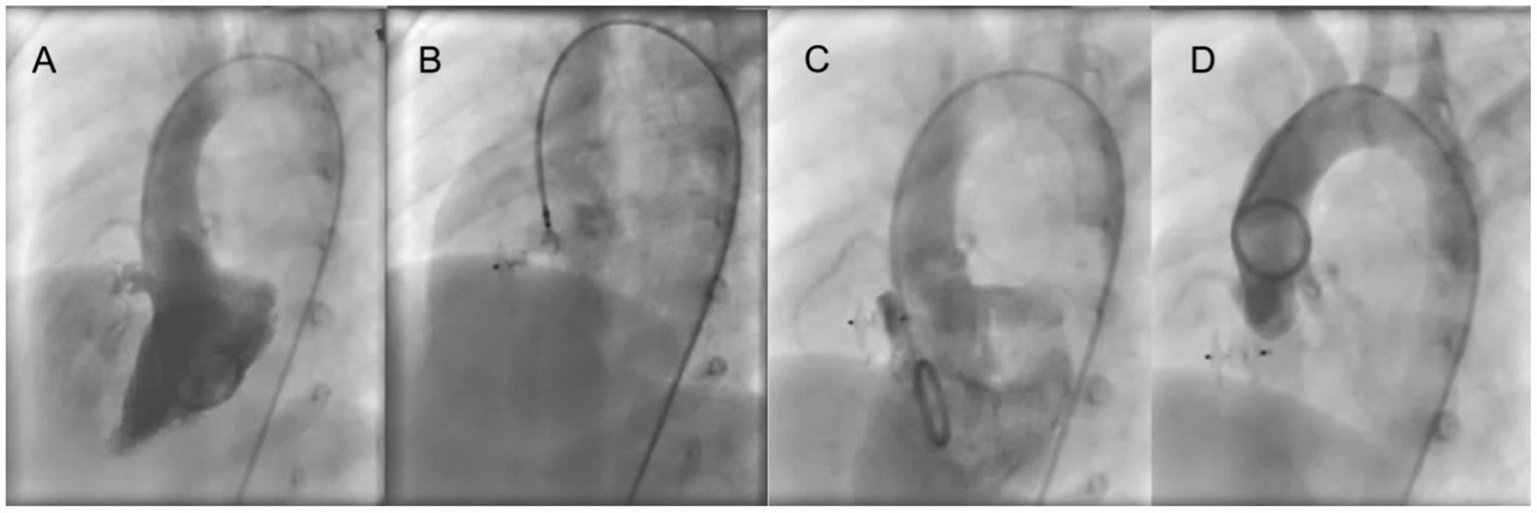
ADO II is used to close an aneurysmal pmVSD. **(A)** Left ventricular angiography shows an aneurysmal shunt jet with 3 outlets. **(B)** Before occluder release, the left disk is pressed into the left ventricle through the delivery sheath and left ventricular angiography is performed to confirm that the left disc is under the aortic valve. Also, transthoracic echocardiography is performed to determine if the left disc is causing aortic regurgitation. **(C)** Left ventricular angiography after occluder release. **(D)** Ascending aorta angiography after occluder release. pmVSD, perimembranous ventricular septal defect.

The catheterization procedure was performed under general anesthesia in all patients. Intravenous heparin (100 U/kg) was given after puncture of the femoral vein and artery, and then right and left cardiac catheterization was performed with calculation of flows and resistances. Left ventricular angiography was performed in long axial oblique view (60° left anterior oblique/20° cranial) (Figures 1A, 2A). The diameter of the device was usually 1–2 mm larger than the largest defect diameter, as measured by angiography. A partly cut pigtail size 5 Fr was used to pass through the defect via the femoral artery. A 0.035-inch hydrophilic guidewire was passed across the defect into the right ventricle and then into the pulmonary artery or superior vena cava. The long delivery sheath (Amplatzer TorqVue Low Profile Delivery System, 5Fr or 4Fr) was advanced to the right ventricle via the guidewire. An occluder was advanced into the delivery sheath and then positioned on the defect. The waist of the occluder was centered in the right ventricular outlet orifice of the defect/aneurysmal pouch, and the left disc was pushed to bring it close to the ventricular septum. Before release of the occluder, left ventricular angiography was performed by hand-pushing contrast agent through the delivery sheath to identify appropriate device position and complete occlusion (Figures 1B, 2B), and then TTE was performed to detect residual shunt and ensure the regular function of the aortic and tricuspid valves. Angiography in left ventricular and ascending aorta was performed after occluder release to confirm complete occlusion and any new-onset aortic valve regurgitation (AR) (Figures 1C,D, 2C,D).

### Post-intervention Treatment and Follow-Up

Following the procedure, all patients underwent clinical examination, ECG monitoring, ECG, chest X-rays, TTE, and 24-h Holter monitoring before discharge. Platelet anti-aggregation therapy with aspirin 3–5 mg/kg/day p.o. was prescribed for 6 months. Follow-up visits were arranged for 1, 3, 6 and 12 months after the procedure and yearly thereafter. Each follow-up visit included physical examinations, ECG, chest X-ray and TTE. Follow-up data were obtained from all patients with successfully pmVSD closure using ADO II. The follow-up data were available until December 2019.

### Statistical Analysis

SPSS 24.0 (SPSS, USA) and GraphPad Prism 7.0 (GraphPad Software, USA) were employed for data analysis. Data are presented as the mean ± standard deviation (SD), and comparisons were performed by Student's *t-*test. *P* < 0.05 indicated statistical significance.

## Results

In 102 of 103 patients, the defects were successfully closed (99.0%) through a retrograde route via the femoral artery. One ADO II was used in each patient, and 98.0% of the waist length was 4 mm (the basic transcatheter closure data are shown in Table 2). Successful transcatheter closure was performed in one single catheter procedure in one patient with a combined atrial septal defect (ASD) and three patients with combined patent ductus arteriosus (PDA). The complete closure rate was 89.2% 24 h after the procedure and 98% at the last follow-up. The TTE within 24h after procedure showed that the left atrial diameter decreased from (2.35 ± 0.38) cm to (2.20 ± 0.32) cm, and the left ventricular end-diastolic diameter decreased from (3.71 ± 0.51) cm to (3.49 ± 0.42) cm, both of which were statistically significant (*P* = 0.000 for both).

**Table 2:** Procedure data.

**Variables**	**Values**
**Without aneurysm**	
Defect diameter (echo)	3.16 ± 0.63 mm (2.0–3.9)
Defect diameter (angio)	1.78 ± 0.27 mm (1.5–2.2)
**With aneurysm**	
Inlet diameter (echo)	5.73 ± 1.32 mm (2.8–14)
Inlet diameter (angio)	5.89 ± 2.91 mm (3.0–18)
One outlet diameter (echo, 62)	2.59 ± 0.64 mm (1.5–4.3)
One outlet diameter (angio, 59)	1.97 ± 0.52 mm (1.5–4.0)
Larger diameter of two outlets (echo, 20)	2.27 ± 0.40 mm (1.8–3.0)
Larger diameter of two outlets (angio, 20)	2.07 ± 0.50 mm (1.5–3.0)
Larger diameter of over two outlets (echo, 10)	2.54 ± 0.54 mm (1.5–3.2)
Larger diameter of over two outlets (angio, 13)	2.02 ± 0.40 mm (1.5–3.0)
Qp/Qs	1.51 ± 0.14 (1.2–1.8)
Fluoroscopy time	11.33 ± 10.50 min (2.15–73)
Radiation dosage	126.99 ± 127.46 mGy (20–775)
**ADO II size (mm)**	
3–4	6 (5.9%)
4–4	21 (20.6%)
5–4	42 (41.1%)
6–4	31 (30.4%)
5–6	1 (1.0%)
6–6	1 (1.0%)

*Values are mean ± SD or n (%). ADO II, Amplatzer Duct Occluder II*.

Reason for failure: In one 3-year-old girl, left ventricular angiography showed the inlet diameter was 9 mm with two outlets away from the aortic valve. The larger outlet diameter was 2 mm. We prepared one 6–4 mm ADO II device, but the patient developed third degree AVB when the 5 Fr delivery sheath was advanced to the right ventricle, which resolved upon removal of the sheath. She underwent successful surgical VSD repair 1 week later.

### Complications

No hemolysis, vascular complications, thrombosis, device embolization, AVB, or death occurred.

### Residual Shunt

TTE showed a residual shunt in 11 patients 24 h after the procedure, with 9 cases of trivial (≤1 mm). At the last follow-up, most of residual shunts (9 of 11 cases) disappeared, only leaving 2 cases with trivial (Table 3).

**Table 3:** Residual shunt.

	**24 h after procedure**	**Last follow-up**
≤1 mm	9	2
1–2 mm	1	0
2–3 mm	1	0

### Valve Regurgitation

There was one patient with mild AR and 8 patients with mild tricuspid regurgitation (TR) before the procedure. No significant increases in AR or TR occurred during or after the procedure. AR in one patient and TR in 6 patients disappeared, and TR in 2 patients relieved during follow-up.

One patient developed new mild AR, and 9 patients developed new mild TR after the procedure. TR in 5 patients disappeared, AR in one patient and TR in 4 patients relieved during follow-up.

### Arrhythmia

One patient developed intermittent complete left bundle branch block (CLBBB) on the 4th day after the procedure and recovered sinus rhythm 2 days later after administration of intravenous dexamethasone (0.5 mg/kg). ECG and 24-h Holter monitoring were normal during the 2-year follow-up. Three patients developed complete right bundle branch block (CRBBB) after the procedure, of which one case reverted to incomplete right bundle branch block and the other two CRBBB persisted during follow-up. There was no incidence of AVB after the procedure or during follow-up.

### Follow-Up

The median duration of follow-up was 36 months (range 6–92 months), and the follow-up rate was 100%. No occluder embolization, acute cardiac or vascular perforation, high-grade or third degree AVB, CLBBB, severe valvular regurgitation requiring surgery, occluder-related endocarditis, severe hemolysis requiring surgical removal of the occluder, death or other serious complications occurred during follow-up.

## Discussion

In the past 20 years, with advances in interventional catheterisation techniques and development of devices, interventional closure of VSD has been widely used in clinical practice. Santhanam et al. (7) concluded in 2018 that in 54 articles from 2002 to 2017, 6,762 patients with pmVSD had an average closure success rate of 97.8%, and the incidence of serious complications was 1.1%. VSD (especially pmVSD) has complex surrounding structures and tissues, and the conduction system passes through that region. Regardless of surgical operation or interventional closure, abnormal cardiac conduction can occur after the procedure. Second, if the closure device was selected improperly, it may affect the abnormal closure of AR and TR, resulting in valve stenosis or regurgitation. At present, there are many conventional choices of intervention closure devices for pmVSD, such as symmetric, eccentric, and thin-waist VSD occluders (8–10). If the distance from the defect to the aortic valve was <2 mm, eccentric occluder was usually selected. Otherwise, other occluders were used. However, if the left disk of device can be placed within the aneurysmal tissue, a non-eccentric occluder may be used even if the distance from the defect to the aortic valve was <2 mm. The thin-waist occluder was mostly used to close a larger aneurysmal pmVSD with multiple outlets. However, there is no one perfect occluder with good performance and low complication rate for the interventional closure of all pmVSD. The performers choose the closure device according to the defect shape and their experience. The Amplatzer Duct Occluder I (ADO I) and spring coil can also be chosen for some special pmVSD (2, 11, 12).

Severe arrhythmia after transcatheter closure of pmVSD is one of the most serious complications, especially cAVB and CLBBB. cAVB has been reported to be the most significant complication in both the early phase and follow-up period, with a pooled incidence rate of 1.1% in total of 6,762 patients (7). Previously reported occurrence rates of cAVB using eccentric and symmetric VSD occluders varied from 1.7 to 5.8% (2, 3, 13) and 0.6 to 1.4% (13–15), respectively. The incidence of cAVB after transcatheter closure of pmVSD using ADO I is about 1.3–1.4% in recent studies (11, 12). Wang et al. (15) used symmetric VSD occluders to perform transcatheter closure of pmVSD in 2,114 children, and 57 cases developed CLBBB with an incidence of 2.7%. It is generally believed that cAVB in the early period after the procedure may be caused by transient myocardial edema or the compression of the atrioventricular node by the occluder (13, 16, 17). It has been reported that cAVB and CLBBB appeared early post-procedure can partially or completely relieve after steroid treatment (2, 15, 16), but some studies have noted that such conditions are likely to recur in long-term follow-up (13, 15, 16, 18). Corticosteroid therapy can reduce acute inflammatory edema, but the compression on the conduction tissue by the occluder cannot be relieved, and can probably increase over time due to the shape memory of the device. Therefore, such arrhythmias are likely to recur during follow-up. The clinical and experimental studies (15, 19) have confirmed that CLBBB can induce abnormal left ventricular contraction and damage global left ventricular function, which might cause progressive left ventricular remodeling and heart failure. In addition to arrhythmia in the early period, some patients will have late-onset cAVB and CLBBB in the middle- and long-term period, and their risk factors are similar to those of early-onset conditions (17). Accordingly, careful monitoring of the heart rhythm remains mandatory throughout follow-up. In those with late-onset and recurrent arrhythmias, it appears more difficult to restore normal conduction, and some patients might need to be treated with an implanted permanent pacemaker or cardiac resynchronization therapy (13–15, 20). Therefore, extensive pmVSD closure using no matter symmetric or eccentric VSD occluder has been limited by the unacceptable high rate of cAVB and CLBBB.

ADO II is a second generation occluder manufactured by Amplatzer for the closure of PDA, and it has been used for VSD closure since 2011 (21). Generally, the choice of ADO II in pmVSD closure is mainly based on two aspects: (1) Because the wire of ADO II is thin, it is softer than the VSD occluder and ADO I and has better compliance of the disc, so it can reduce the impact on the aortic valve and the occurrence of AR as much as possible. (2) The waist of the ADO II can easily extend, which may reduce the compression or friction on the conduction tissue around VSD and alleviate myocardial edema so that the incidence of conduction abnormalities after the procedure can be reduced. In comparison with pmVSD occluders, ADO II is smaller, softer and more flexible device that can be released, retrieved, and repositioned lightly through a thinner delivery sheath (22). A few studies have shown that the use of ADO II in pmVSD closure is safe and effective, with a success rate of 93.5–100% (4–6). Kanaan et al. (4) followed up 29 patients for 0.6–67 months without serious complications. Pamukcu et al. (5) followed up 49 patients for up to 66 months, and one patient presented cAVB and a large amount of pericardial effusion 6 months after the procedure and was implanted with a permanent pacemaker. In our cohort, all adverse events were minor and there were no serious complications.

ADO II was selected in this study mainly to close the tube type with a longer sinus tract or perimembranous aneurysmatic defects. Especially for the former, the waist length of the common VSD occluder was shorter, which could not meet the needs for closure. There have been reports of using ADO I to close that type of pmVSD (11, 12). The waist of ADO II is easily extendable and softer than that of ADO I. So the effect of the occluder on the surrounding structure was decreased, and the likelihood of long-term damage to the tissues surrounding the VSD (such as the tricuspid chordae tendineae) was also reduced. Therefore, ADO II is the more appropriate device for this type of VSD.

In most of the previous reports, the establishment of an arteriovenous track was needed and ADO II was released through the anterograde insertion route via the femoral vein, but transcatheter retrograde closure via the femoral artery was rare. Currently, the delivery sheath of common VSD occluder is 6 Fr or thicker. The insertion from the femoral artery can cause great damage to the artery, and the thicker sheath may increase the chance of injury to the aortic valve. Thus, pmVSD is usually closed through the anterograde insertion route via the femoral vein at present. However, anterograde occlusion requires the establishment of an arteriovenous track what is complicated. Sometimes, as a result of the insertion of guide wire into the space between the chordae tendoneae of the tricuspid valve or improper selection of the occluder, the track might need to be reconstructed repeatedly, which will prolong the procedure time and increase the associated risk. The incidence of AR after pmVSD occlusion was reported to be around 3.3–11.0%, and the incidence of TR was 5.4–40% (23). ADO II can be incorporated into a tiny 4–5 Fr long sheath and released both through the anterograde route via the femoral vein and through the retrograde route via the femoral artery. The release of ADO II via the femoral vein also needs to establish arteriovenous track. It has been reported that retrograde release via the femoral artery also requires the establishment of an arteriovenous track (5). Koneti et al. (6) ever used a 5 Fr or 6 Fr right coronary artery guiding catheter with side holes as the delivery catheter to successfully release ADO II via the femoral artery in 57 patients. In our study, all the occluders were released retrograde through the femoral artery without the establishment of an arteriovenous track. To our knowledge, our study includes the largest series of pediatric patients who underwent pmVSD closure using this approach with long-term follow-up. This procedure was easier than the anterograde release approach and significantly reduced the fluoroscopy time. More importantly, it could prevent injury to the tricuspid valve and aortic valve caused by the catheter and guide wire during the establishment of the arteriovenous track. Meanwhile, the thinner sheath caused less harm to the femoral artery especially for infant, and may decrease damage to the aortic valve. When we released the left disc, we needed to push the delivery sheath into the left ventricle to avoid affecting the closure of the aortic valve after release. Previous research required that the upper margin of defect to aortic valve distance should be at least 3 mm. However, we found that for the larger perimembranous aneurysmatic defects, we may choose to put the whole left disc or at least the upper edge of the left disc into the aneurysm to avoid AR even if the distance from the upper edge of the inlet to the aortic valve was <3 mm. As the waist was longer, we tried to release the waist into the aneurysm to prevent the left disc from protruding more into the left ventricle or the right disc protruding more into the right ventricle, which could result in left or right ventricular outflow tract stenosis and tricuspid incompetence. In our study, the incidence of AR and TR post-procedure was 1 and 8.8%, respectively. They were all mild, and relieved or disappeared during follow-up.

At present, using ADO II to close pmVSD is gradually becoming more common, and the incidence of complications is relatively lower. The advantages of ADO II are expected to reduce or even eliminate the shortcoming of traditional pmVSD occluders. However, this method is still considered an “off-label” approach and lacks a study with a large sample size and long-term follow-up. The potential benefits of this method for reducing heart blocks need to be demonstrated in longer follow-up. Studies with large samples and long durations should be conducted to further confirm the safety and benefits.

Although our research has shown the advantages of using ADO II to close pmVSD, its clinical indications are still strictly limited. The two disks are 6 mm larger than the waist (3 mm on each side), which generally requires that the distance from the upper edge of the defect to the aortic valve is 3 mm at least. The maximum waist diameter of ADO II is only 6 mm at present, so the larger defects cannot be closed using ADO II. Because of its large disc and long waist, its position should be noticed when release to avoid causing valvular inadequacy and outflow obstruction. This study also has drawbacks. First, the research was performed in a single center and was a non-randomized study, and long-term follow-up is required to further evaluate the safety and efficacy of ADO II compared with the outcomes of other conventional occluders for pmVSD. Second, these findings are applicable only in larger heart centers, and a multicentre prospective study with a large sample is needed.

## Conclusion

Transcatheter retrograde closure of pmVSD via the femoral artery with ADO II in children is safe, feasible and effective in selected patients. ADO II has unique advantages in transcatheter closure of some types of pmVSD. The approach through the femoral artery for retrograde closure is simple, and has a high success rate and a low incidence of complications during long-term follow-up. ADO II has become good alternative devices for percutaneous pmVSD closure.

## Data Availability Statement

The original contributions presented in the study are included in the article/Supplementary Material, further inquiries can be directed to the corresponding author/s.

## Ethics Statement

The studies involving human participants were reviewed and approved by the Ethics Committee of Shandong Provincial Hospital Affiliated to Shandong First Medical University. Written informed consent to participate in this study was provided by the participants' legal guardian/next of kin.

## Author Contributions

DJ and JZ designed the study and performed the research. YF, LZ, YY, JL, and JW performed the research and analyzed the data. DJ wrote the manuscript. BH and JZ supervised the study. All authors contributed to the article and approved the submitted version.

## Conflict of Interest

The authors declare that the research was conducted in the absence of any commercial or financial relationships that could be construed as a potential conflict of interest.
